# Aging: Molecular Pathways and Implications on the Cardiovascular System

**DOI:** 10.1155/2017/7941563

**Published:** 2017-08-09

**Authors:** Arthur José Pontes Oliveira de Almeida, Thaís Porto Ribeiro, Isac Almeida de Medeiros

**Affiliations:** Departamento de Ciências Farmacêuticas/Centro de Ciências da Saúde, Universidade Federal da Paraíba, Cidade Universitária-Campus I, Caixa Postal 5009, 58.051-970 João Pessoa, PB, Brazil

## Abstract

The world's population over 60 years is growing rapidly, reaching 22% of the global population in the next decades. Despite the increase in global longevity, individual healthspan needs to follow this growth. Several diseases have their prevalence increased by age, such as cardiovascular diseases, the leading cause of morbidity and mortality worldwide. Understanding the aging biology mechanisms is fundamental to the pursuit of cardiovascular health. In this way, aging is characterized by a gradual decline in physiological functions, involving the increased number in senescent cells into the body. Several pathways lead to senescence, including oxidative stress and persistent inflammation, as well as energy failure such as mitochondrial dysfunction and deregulated autophagy, being ROS, AMPK, SIRTs, mTOR, IGF-1, and p53 key regulators of the metabolic control, connecting aging to the pathways which drive towards diseases. In addition, senescence can be induced by cellular replication, which resulted from telomere shortening. Taken together, it is possible to draw a common pathway unifying aging to cardiovascular diseases, and the central point of this process, senescence, can be the target for new therapies, which may result in the healthspan matching the lifespan.

## 1. Introduction

**Figure 1:**
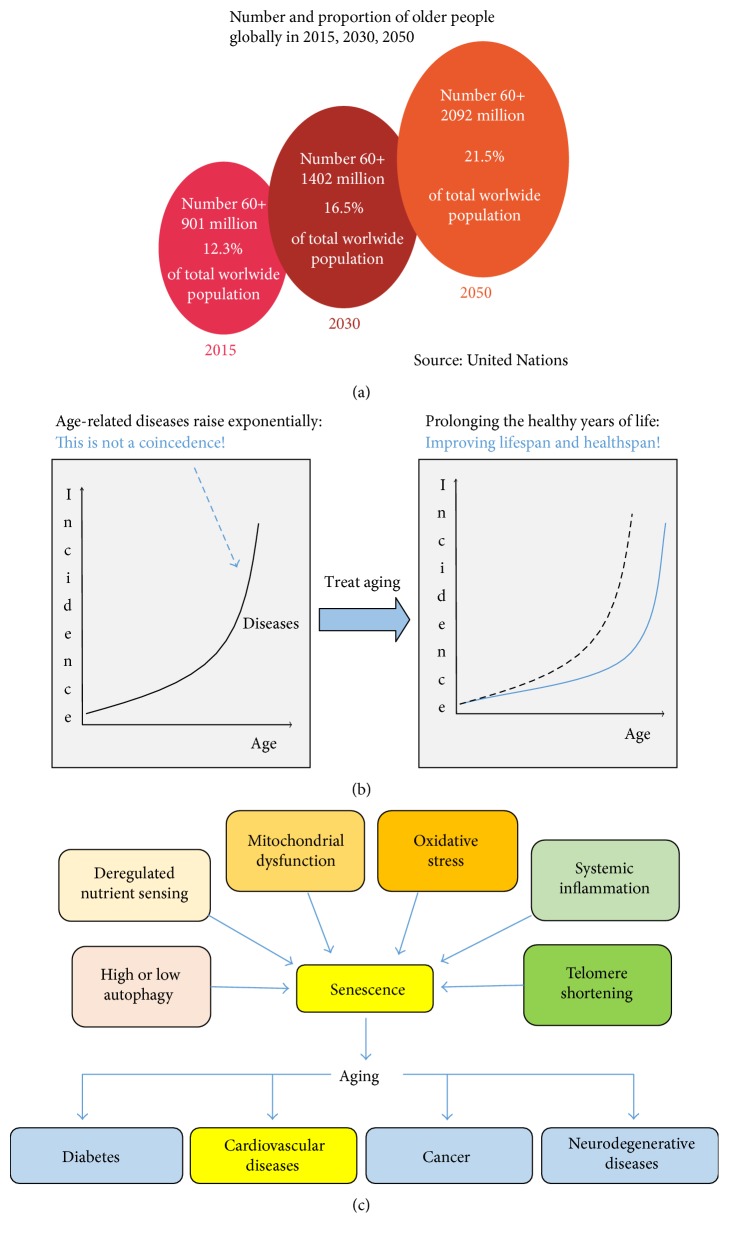
Aging and health. (a) The global population will increase from 12% in 2015 to almost 22% in 2050 [1]. (b) Despite the increase in lifespan, the individual healthspan does not follow this growth, which means that targeting aging with new therapies is essential to minimize the onset of aging-related diseases. (c) At the cellular level, aging is characterized by an increase of senescent cells in the organism, caused by several factors, including oxidative stress, systemic inflammation, mitochondrial dysfunction, deregulated nutrient sensitivity, autophagy dysfunction, and telomere shortening. The same mechanisms that lead to aging drive towards age-related diseases, in particular, the cardiovascular diseases, the major cause of death in the worldwide.

According to the United Nations, the worldwide population over 60 years will grow exponentially over the next decades, rising from 12% in 2015 to 22% in 2050 (Figure 1(a)) [1]. Despite the increase of lifespan, individuals do not necessarily present an improvement in their quality of life (Figure 1(b)). Diseases such as cancer, diabetes, and neurodegenerative and cardiovascular diseases (CVDs) have their prevalence increased with age, being known as age-related diseases. In 2012, 68% of deaths were associated with these diseases, highlighting to CVDs, corresponding to 46% of this total [2].

Aging is a universal and multifactorial process characterized by a gradual decline of physiological functions, occurring at the molecular, cellular, and tissue levels [3], which involve a series of mechanisms such as deregulated autophagy, mitochondrial dysfunction, telomere shortening, oxidative stress, systemic inflammation, and metabolism dysfunction [4, 5]. The deregulation of these pathways leads the cell to a senescent state, which contributes to aging phenotype and, eventually, driving towards age-related diseases (Figure 1(c)). Although many theories have been proposed to explain the aging process, neither of them appears to be fully satisfactory.

Therefore, this review draws an integrated approach to aging, addressing the mechanisms that lead the cell to senescence and how this process can contribute to aging and age-related diseases, with emphasis on the cardiovascular system.

## 2. Senescence: Cellular Retirement

Senescence is the cellular state characterized by cell cycle arrest, usually in G1 phase, but the cells remain metabolically active [6]. Senescent cells secrete a variety of proinflammatory cytokines, interleukins, and growth factors, which has been reported as “secretory phenotype associated with senescence” (SASP) [7].

Senescent cells are usually removed by the immune system; however, in consequence of immunosenescence, they start to accumulate with age [8, 9]. It is believed that increases in proinflammatory mediators are initially a mechanism of “cleaning” the senescent cells, but with immunosenescence, the stimulus generated by the senescent cells are not able to recruit enough functional cells of the immune system, a long-term process that play a negative effect on aging and age-related diseases [10, 11]. Furthermore, there is a limit made by senescence in stem cell lineages, “stem cell exhaustion,” resulting in a decreased regenerative potential [12, 13]. These two hallmarks, accumulation of senescent cells and loss in function of regenerative lineages, contribute to aging simultaneously.

Two major pathways control the senescent state: p53/p21 and p16/pRB. Both pathways are complex and have several regulators; however, in the cardiovascular system, they are still not totally clarified [3, 14, 15]. In response to DNA-damage response (DDR), p53 is stimulated and induces p21 expression, a cyclin-dependent kinase (CDK) inhibitor. In consequence of CDK activity suppression, the retinoblastoma protein (pRB) is activated. The p16, another CDK inhibitor, also prevents the pRB phosphorylation, leading to pRB inactivation [6, 15, 16]. Thereby, pRB plays a central role in the senescence and its activity is mainly attributed to its ability to bind and inactivate the E2F family of transcription factors, which induces cell cycle proteins and DNA replication factors required for cell growth [16]. In this way, there is a reciprocal regulation between the p53/p21 and p16/pRB signalling; however, these pathways can induce senescence independently [6]. Indeed, “cleaning” naturally occurring p16 positive cells improves healthspan, which presents several benefits on the cardiovascular system [17].

**Figure 2:**
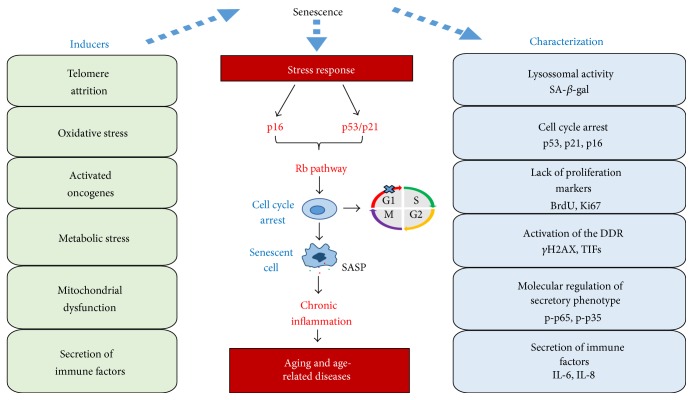
Senescence and aging. Aging is characterized by senescent cell accumulation into the body. Senescence can be achieved replicatively or induced by stress. Once activated, the p16 and p53/p21 pathways converge with each other, regulating the Rb mechanism, leading to cell cycle arrest, and consequently, the senescence. This results in the release of cytokines and chemokines, driving towards a systemic inflammatory condition that lead to aging and age-related diseases. The senescent cells are characterized by a high lysosomal *β*-galactosidase activity and, in association with others characteristic factors, consist the gold standard for the senescence characterization.

Morphologically, the senescent cells are characterized by the increase in volume, and if adherent, they adopt a flattened morphology; however, there is no marker exclusive to a senescent state [3]. The first marker to be used was the detection of senescence associated with *β*-galactosidase (SA-*β*-gal) activity [18], which actually indicates increased lysosomal activity of *β*-galactosidase [19]. Recently, several molecular markers were developed and their association with SA-*β*-gal is the gold standard to confirm the senescent stage in vascular cells [20]. Such markers represent the cell cycle arrest (p16, p21, and p53), lack of proliferation markers (Ki67, BrdU), expression of secretion factors (IL-6, IL-8), activation of secretory phenotype-regulating pathways (p-p65 or p-p38), changes in chromatin (HP1, Hira), and activation of the DDR (*γ*H2AX, TIFs) (Figure 2) [21, 22].

Several factors lead to senescence, and one of them is the cellular division, with telomere shortening, called replicative senescence [23, 24]. In endothelial and smooth muscle cells, senescence can also be induced by stress, such as oxidative stress and inflammation leading to DNA damage, activation of oncogenes, and changes in chromatin [14, 21]. Another route that leads to senescence is the mitochondrial dysfunction, a process that decreases cellular energy supply, leading the cell to decrease its metabolic activity [25, 26]. In addition, deficiency in the pathways of autophagy also leads the cell to the senescence through the accumulation of cellular “waste,” which is toxic to the cell, including vascular cells [27].

The raising in the number of cardiac, muscular, endothelial, and endothelial progenitor senescent cells has been associated with cardiovascular dysfunction, leading to the progress of several diseases, such as hypertension, atherosclerosis, heart failure, and stroke. Therefore, therapies aimed at reversing or delaying the senescence process have been proposed for the treatment of these diseases [17, 28–31].

## 3. Telomeres: The Biological Clock

**Figure 3:**
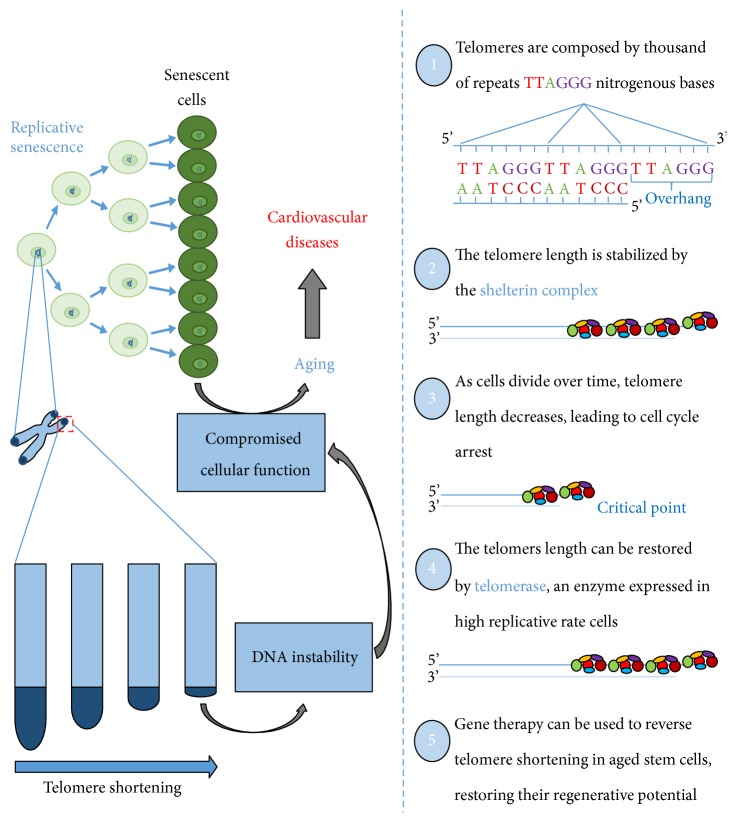
Role and function of telomeres in DNA protection. After each cell division, each chromosome loses a part of its telomeres, a region characterized by thousands of repeated sequences of nitrogenous bases. At a critical point, cells with shortened telomeres stop to divide, leading to senescence and resulting in aging and CVDs. Cells with high replicative rates such as stem cell lineages express telomerase, an enzyme capable of reversing telomere shortening. This enzyme plays a key role in the development of new therapies that aim to slow or reverse the aging process.

One of the hallmarks of molecular aging is the telomere shortening with the advent of age [4]. Telomeres, known as the biological clock, comprise thousands of nucleotide sequences at the end of each chromosome. In the 3′ side, the sequence corresponds to TTAGGG (9–15 kb, in humans) [32]. In somatic cells, after each cell division, part of these bases is lost in the process, promoting telomere shortening [23]. Thus, it is estimated a finite number of cellular divisions and, after that, cells become senescent (Figure 3) [33].

Associated with telomeres, there is a shelterin complex formed by proteins and transcription factors. This complex comprises a set of six subunits with distinct functions, which has essential participation for chromosome protection [34]. They are telomere repeat-binding factor 1 (TRF1), telomere repeat-binding factor 2 (TRF2), repressor-activator protein 1 (RAP1), TRF1- and TRF2-interacting nuclear protein 2 (TIN2), tripeptidyl-peptidase 1 (TPP1), and protection of telomere 1 (POT1) [35]. TRF1 and TRF2 bind directly to the double-stranded telomeric repeats, while POT1 recognizes the telomeric strand in the 3′ branch. TIN2 binds to TRF1 and TRF2. TIN2 also recruit the TPP1-POT1 heterodimer, reducing different shelterins to organize the final portion of the telomeres. RAP1 is recruited to the telomeres by TRF2. In addition, RAP1 can also bind along chromosomal arms regulating gene transcription [36].

The telomeres participate in the maintenance of the genome and promote stability in the replication process, avoiding undesirable recombination and chromosomal fusion [37, 38]. When the critical telomere size is reached, the proteins cannot be recruited to maintain the T-loop nucleotide sequences. Then, the DNA repair system activates cellular checkpoints [39, 40]. Two checkpoints have already been identified that limit cell life in response to telomeres dysfunction: the first checkpoint (M1, the first stage of mortality) is characterized by a complete cell cycle arrest, known as senescence, and it is dependent on p53 activation [32]. Cells mutated in the p53 gene may continue to divide even when the critical size of the telomeres was reached [34, 41]. If the cell continues to divide and, consequently, the telomeres continue to decrease in size, a new checkpoint is activated (M2, the second stage of mortality), called the crisis. This point is independent of p53 and is characterized by massive chromosomal instability and cell death [42].

In some cellular lineages, such as stem cells, telomere shortening can be restored by the enzyme telomerase reverse transcriptase (TERT), together with its RNA component (TERC) [43]. Both are regulated by the shelterin complex [44]. The ability of embryogenic or induced pluripotent stem cells (iPSC) to replicate indefinitely is due to a high expression of functional TERT and TERC in these cell populations [45, 46]. Several studies have reported that inducing TERT activity in somatic cells reverses several characteristics of aging, such as senescence [47, 48], which leads to cardioprotection [49]. In addition, hearts expressing TERT showed attenuated cardiac dilation, improved ventricular function, and smaller infarct scars concomitant with increased mouse survival by 17% compared with controls [50].

Furthermore, telomere shortening in circulating lymphocytes, used as an indirect marker of circulating progenitor cells, has been identified as an early-onset alarm for CVDs [51].

Cardiac telomerase activity is detectable at the earliest stages of life and is downregulated in an adult rat myocardium [52, 53]. Recently, Richardson and colleagues showed a natural expression of telomerase functionally important in adult mammalian hearts [54], which could be targeted for cardiovascular regeneration.

Therefore, there is a great evidence that combating telomere shortening has beneficial effects on the cardiovascular system, through slowing or even reversing cellular senescence [50, 55, 56].

## 4. The Role of ROS and Oxidative Stress: A Necessary Evil

**Figure 4:**
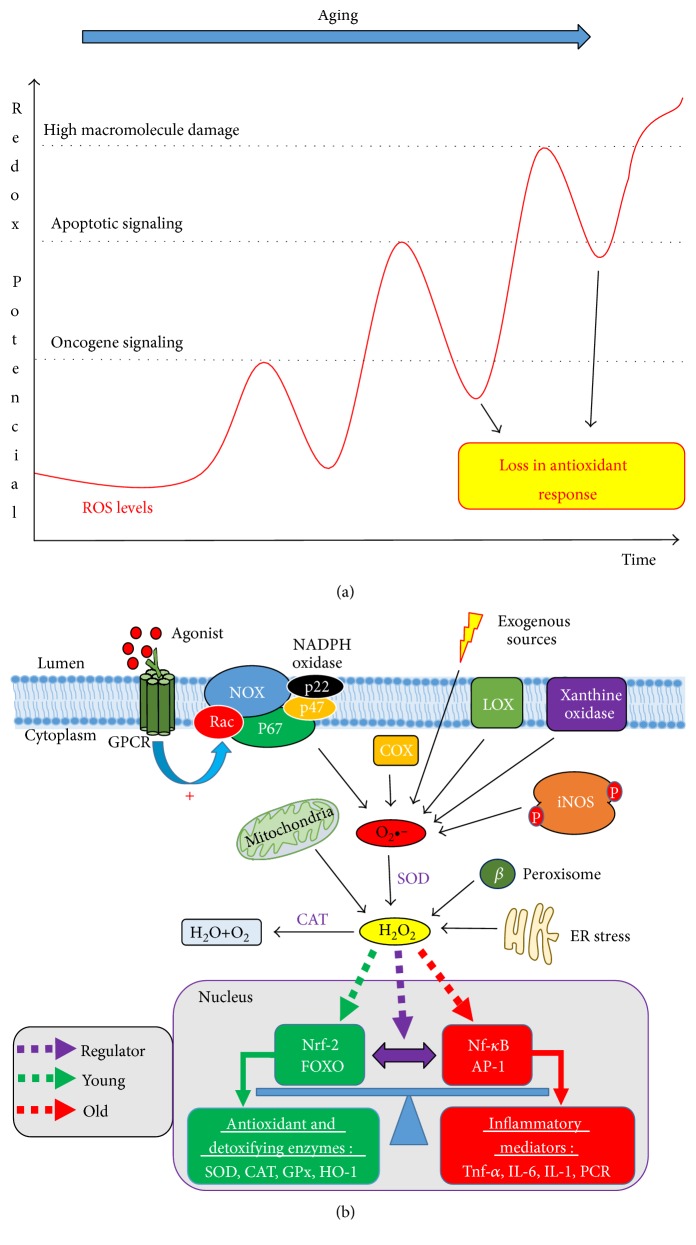
Redox potential controls cell fate. One of the hallmarks of aging is the increase in ROS levels production. New approaches define this increase as a compensatory cellular response with the original purpose to maintain cellular homeostasis and, from a certain limit, as a factor that aggravates aging. (a) The increase in ROS levels, first as a factor that activates survival pathways, continues to increase as a consequence of the deficiency in the antioxidant system, generating other cellular responses such as apoptosis, with a failure in apoptotic signalling, and driving towards severe cellular damage, such as necrosis. (b) Several sources of ROS contribute to the increase of redox potential, a factor that shifts the balance to the transcription of pro-inflammatory factors, while the antioxidant genes are silenced, connecting ROS and inflammation to aging.

According to the free radical theory of aging proposed by Harman in 1956, ROS leads to oxidative damage in cellular biomolecules, contributing to the decline of physiological function with aging [57]. Although a series of reviews and evidences reports the deleterious effects of ROS in aging [58, 59], recent studies on long-lived models and genetically altered animals challenge the role of ROS in aging [60]. In this way, ROS seems to have a double effect, initially, as an activator of a homeostatic compensatory response that increases with age in order to maintain survival through activation of various defence mechanisms plus stimulating cellular proliferation and, from a certain limit, as a factor that, instead of alleviating, aggravates the damages associated with aging (Figure 4(a)) [61, 62].

There are several sources of ROS in mammals, including mitochondrial respiration, cyclooxygenase and lipoxygenase, cytochrome p450s, xanthine oxidase, NADPH oxidase, NO synthase, peroxidase, endoplasmic reticulum, and other hemoproteins [63, 64]. Many ROS species have unpaired electrons, called free radicals. In this group, these include superoxide anion (O_2_^−^•), hydroxyl radical (HO•), nitric oxide (NO•), and lipid radicals. Other reactive oxygen species such as hydrogen peroxide (H_2_O_2_), peroxynitrite (ONOO^−^), and hypochlorous acid (HOCl) are not free radicals but have oxidizing effects that contribute to oxidative stress [65, 66].

The basal balance in ROS levels is mediated by the activity of a set of enzymatic and nonenzymatic complexes with the function of cellular detoxification, collectively called antioxidants [67]. Nuclear factor erythroid 2-related factor 2 (Nrf-2), a transcription factor, is the major regulator of the antioxidant enzymatic system in the vasculature, including transcription of antioxidant enzymes and phase II detoxifiers such as superoxide dismutase (SOD), catalase (CAT), glutathione peroxidase (GPx), glutathione reductase (GR), hemeoxygenase-1 (HO-1), and NAD(P)H quinone oxidoreductase-1 (NQO1). Taken together, this system is the major defence system that counteract ROS production in vivo [68, 69].

An imbalance to the prooxidant side leads to the physiological status known as oxidative stress, which has been linked to impaired vascular function [70].

NADPH oxidase (Nox) is an important source of ROS on the cardiovascular system [71]. There are seven Nox isoforms: Nox1, Nox2, Nox3, Nox4, Nox5, Duox1, and Duox2. All Nox are transmembrane proteins that have a catalytic site (Nox) and a regulatory protein complex [72]. Isoforms 1, 2, 4, and 5 are expressed in various tissues including the heart and vessels. Nox2 and Nox4 are superexpressed in the vascular tissue of old mice [73]. The prototype of the group is the Nox2 which is composed of 6 subunits: p47phox, p67phox, p40phox, and Rac1/2 which are cytosolic regulatory proteins; p22phox which is a membrane regulatory protein; and gp91phox which is a catalytic subunit present in the membrane [74].

The Nox complex is upregulated by TNF-*α* [75] and also by the activation of AT1 receptor by angiotensin II [76]. Thus, in the increase in ROS production, Nrf-2 begins to have its activity inhibited by the crosstalk with Nf-kB, which is responsible for increasing TNF-*α* levels, generating a vicious cycle [77]. As ROS are produced, TNF-*α* release increases, aggravating oxidative stress. This shift in the expression of Nrf-2 to Nf-kB seems to be gradual, accompanying aging, and directly proportional to the increase in cellular dysfunction (Figure 4(b)).

The main source of ROS during aging is the mitochondria [78]. Harman in 1972, reviewed his theory about free radicals after the discovery that mitochondria turned oxygen into water, a process that, when deficient, results in a high production of superoxide anions, raising mitochondrial ROS (mtROS) levels, which lead to the accumulation of mitochondrial DNA (mtDNA) mutations, driving towards mitochondrial dysfunction, resulting in aging [79]. However, recent evidences involving mtROS using longevity animals modelling reject, at least in part, the original idea of the mitochondrial theory of aging [62]. These pathways conserved from yeast to mammals have been subsequently assessed for their role in regulating longevity, as well as to their roles played in CVDs [80].

Studies with *C. elegans* report that, by deletion of the SOD2 gene, the increase in mtROS seems to prolong lifespan [81]. In *Drosophila*, mtROS from the electron transport chain also appears to have a positive effect on the lifespan [82]. In addition, overexpression of catalase increases resistance to oxidative stress but do not improve lifespan [83]. In worms, antioxidant diets reduce their lifespan [84]. In mice, genetic alterations that increase mtROS and oxidative damage do not accelerate aging, although induce the appearance of various age-related diseases [85]. There is evidence that mtROS and cytosolic ROS have opposite effects, being the cytosolic more toxic to the cell [86].

Therefore, the H_2_O_2_ produced with beneficial propose in the mitochondria, eventually diffuses through the mitochondrial membrane [87], reaching to the cytoplasm contributing to the oxidative stress involved in aging, suggesting that ROS effects are dependent on where they are present and their concentration [86].

The elderly are more susceptible to oxidative stress due to a reduction in the efficiency of their endogenous antioxidant systems. Organs such as the heart, which it has a limiting rate of replication and high levels of oxygen consumption, are particularly sensitive to this phenomenon, which explains, in part, a high prevalence of CVDs in aging [88]. On the other hand, in endothelial cells, ROS derived from NADPH oxidase complex induces in vivo kinase prosurvival via AMPK, plus an additional effect of inducing autophagy, improving the vascular function in aged mice coronary [89]. Thus, this approach can integrate paradoxical concepts about the beneficial, deleterious, or neutral role of ROS in aging.

## 5. Inflammation: A War without an Army

Aging is accompanied by a systemic increase of proinflammatory agents, a phenomenon known as “inflammaging” [90]. Senescent cells have the ability to release proinflammatory agents (ASAP) capable of attracting defence cells, that phagocyted the senescent cells [91, 92]. However, in aging, the exhaustion of stem cells occurs, reducing the regenerative capacity of the organism, as well as the production of functional immunity cells, a term known as immunosenescence, allowing the accumulation of senescent cells into the body, which is related to the onset of cardiovascular diseases [8, 93].

The ASAP components include agents such as tumor necrosis factor-*α* (TNF-*α*), interleukin-6 (IL-6), and IL-1*β* [94]. These proinflammatory agents are mainly regulated by transcription factors sensitive to redox potential, as the activator of protein-1 (AP-1) and nuclear factor kappa B (Nf-kB) [95]. Overproduction of ROS is essential for activating AP-1 and Nf-kB through the stress of kinases such as extracellular signal regulatory kinases (ERKs), c-jun N-terminal kinases (JNKs), p38 mitogen-activated protein kinase (p38 MAPK), protein kinase C (PKC), phosphatidylinositol-4,5 bisphosphate 3-kinase (PI3K), Akt, and Src family kinases (SFK) [96].

This leads to the increased expression of inflammatory target proteins such as matrix metalloproteinase-9 (MMP9), intercellular adhesion molecule-1 (ICAM-1), vascular cell adhesion molecule 1 (VCAM-1), inducible nitric oxide synthase (iNOS), cyclooxygenase-2 (COX-2), and cytosolic phospholipase A2 (cPLA2) and proinflammatory mediators such as the TNF-*α*, IL-1, and IL-6. Many of these inflammatory proteins or their products such as iNOS, COX, and PGE2 are prominent sources of ROS [96–99]. In fact, the presence of these inflammatory biomarkers in aging is related to the endothelial damage, vascular smooth muscle cell (VSMC) proliferation, and matrix remodelling, being associated to the genesis and progression of cardiovascular diseases, such as atherosclerosis and hypertension [100–102]. Moreover, targeting the overexpression of redox-sensitive transcription factor, NF-*κ*B, by anti-inflammatory molecules seems to play positive effects on the prevention of clinical manifestations of vascular aging, the step to cardiovascular disease [103].

Therefore, one of the fundamental features associated with cardiovascular aging is the crosstalk between oxidative stress and inflammation (Figure 4(b)). It is necessary to point out that both processes contribute to the physiological organism defence, and in the young individual, these processes are with their basal functional activity. For the inflammatory signalling stimulation, it is a necessary increase in the redox potential, which is achieved by elevated ROS generation, especially regulated by the mitochondria.

## 6. Mitochondrial Dysfunction: Communication Failure

Mitochondria are considered the cellular “powerhouse,” since they have the ability to generate adenosine triphosphate (ATP) through oxidative phosphorylation (OXPHOS), providing chemical energy for cellular survival and function [25]. In addition, there is evidence that mitochondria play a nonenergetic role in the regulation of metabolism, apoptosis, innate immunity, and cardiovascular aging [104–106].

Despite that most mitochondrial genes were transferred to the nuclear genome, 13 subunits essential for OXPHOS activity remain encoded by mtDNA. The other 76 subunits are encoded by nuclear genome, being synthetized in the cytoplasm and imported to the mitochondria, requiring functional communication between both genomes [107, 108]. This functional interaction is essential for mitochondrial health, and the failure of this communication leads to mitochondrial dysfunction, decreasing ATP synthesis [106]. In this way, the failure in energy status drives towards endothelial dysfunction, plus inflammation, and oxidative stress, being related to vascular remodelling [109]. In addition, mitochondrial dysfunction is associated to chronic oxidative stress in aged vessels and cardiomyocytes, leading to a deregulation of the cardiovascular system [110].

The mitochondrion regulation occurs mainly by peroxisome proliferator-activated receptor-*γ* coactivators *α* and *β* (PGC-1*α* and PGC-1*β*, resp.), which responds to changes in nutrient status, such as the ratio of NAD^+^/NADH and AMP/ATP (regulated through SIRT1 and AMPK, resp.) [111, 112]. The expression of PGC-1*α*/*β* plays a fundamental role in mitochondrial biogenesis, protecting the vascular endothelium and consequently promoting vascular homeostasis [113, 114].

Recently, Gomes and colleagues described a process of mitochondria regulation via HIF-1*α*, independent of PGC-1*α*/*β*, in response to SIRT1 activity, which it is controlled by nuclear NAD^+^ levels. Six hours after induction of the deletion of the SIRT1 gene in myoblasts, HIF-1*α* levels begin to rise, and after 12 hours, loss of mitochondrial homeostasis occurs, although ROS levels only increase after 24 hours of the procedure [115]. This HIF-*α*-mediated ROS in *C. elegans* is the main determinant of lifespan, but the mechanisms involved are still not fully understood [116].

In aging, there is no loss in SIRT1 levels in the body, but NAD^+^ levels decrease with age, leading to a downregulation of SIRT1 activity [117]. As a result, a pseudohypoxic state occurs, decreasing the activity of complexes I, III, IV, and V (encoded by nuclear and mitochondrial genomes) but not complex II (encoded by the nuclear genome) [107]. To recover the activity of complexes I, III, IV, and V, it is necessary to restore mtDNA and nuclear DNA communication, which is achieved by NMN supplementation (NAD^+^ precursor). Treatments that restore NAD^+^ levels have been shown to be beneficial in restoring mitochondrial function and several aspects related to aging in mice [115, 117, 118], indicating that aging is, at least in part, caused by a failure in nuclear-mitochondrial communication, a process that is dependent of energetic cellular balance [112, 115].

Treatments that promote mitochondrial health drive towards an improvement in metabolism and heath aging and are related to several benefits on the cardiovascular system [119–121]. In addition, targeting mitochondria seems to have positive effects on the cardiovascular system [122].

**Figure 5:**
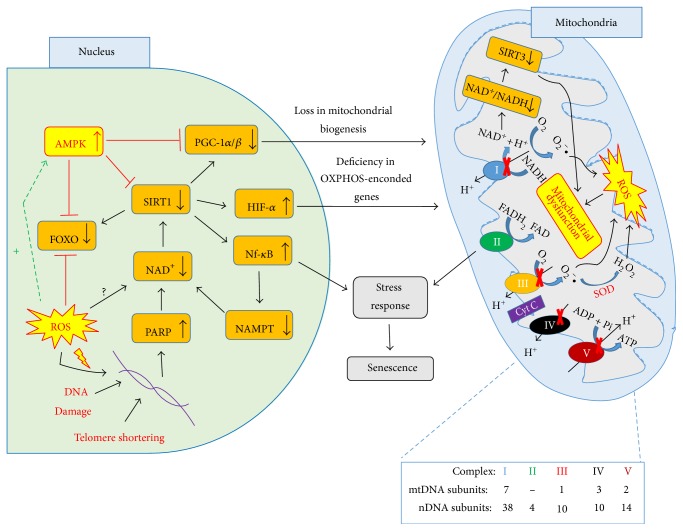
Schematic overview of nucleus-mitochondria communication in aging. Decreased SIRT1 activity due to decreased NAD^+^ levels leads to changes in various gene expression. (1) Decreased PGC-1*α*/*β* levels, leading to decline the mitochondria biogenesis. (2) Increased expression of HIF-*α*, leading to a pseudohypoxia state and, consequently, a missed nucleus-mitochondria communication, driving towards a failure in coding OXPHOS genes. (3) Increased expression of Nf-kB levels, leading to inflammation, plus decreasing NAMPT production, a precursor of NAD^+^. (4) Decreased expression of FOXO, a factor that participates in cytoprotection. These responses are accompanied by increased ROS levels, a factor that activates AMPK, acting together as factors that counteract the decrease of SIRT1 activity. Increasing the ROS levels from a certain limit promote DNA damage, creating a paradoxical effect. In the mitochondria, the failure in the OXPHOS leads to a decrease in energy supply and, combined with oxidative stress, drive towards mitochondrial dysfunction. These factors combined lead to cellular stress and consequently to senescence. The interaction of oxidative stress and NAD^+^ levels are still unclear and may be an important source to understand how redox potential controls cellular energy metabolism.

In a wide perspective, it is possible to identify that a failure in the cellular energy creates a stressful environment that eventually leads to senescence (Figure 5()). The ROS increased with the mission of stimulating survival mechanisms and also promote DNA damage, driving towards aging. It is still not clear whether the relation between ROS and NAD^+^ levels in vascular cells could help to understand the increased redox potential in these cells. Moreover, to maintain the energy status in satisfactory levels, mechanisms that counteract ATP depletion, such as autophagy, play a fundamental role in protecting cells from the energy failure due to mitochondrial dysfunction.

## 7. Autophagy: Cellular Scavengers

Autophagy or “self-eating” refers to the lysosomal degradation process that removes protein aggregates, damaged organelles, toxic substances, and even pathogens [123]. This process is essential to maintain cell integrity and homeostasis by providing metabolites for cell survival under stress conditions [124]. In addition, it helps to maintain cellular energy levels during nutrient limitations through catabolic recycling processes [125].

**Figure 6:**
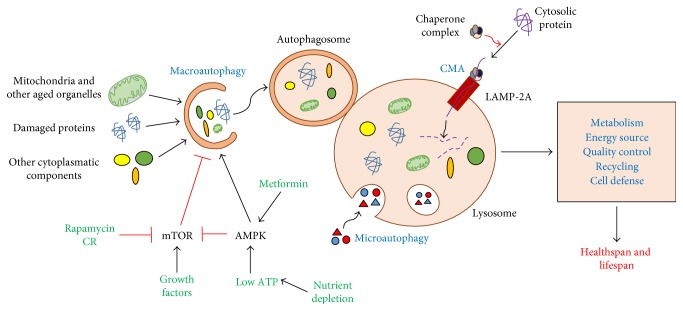
Role of autophagy as cellular scavengers. Autophagy is mainly regulated by two energy sensors: mTOR and AMPK. mTOR is an inhibitor of autophagy and is activated when there are abundant cellular nutrients. AMPK is activated when nutrients deplete, inducing autophagy by inhibiting mTOR, as well as direct activation of autophagy. This mechanism is important for cell “cleaning,” degrading damaged organelles, protein aggregates, and other cellular toxic components. After the formation of the autophagosome, there is fusion with the lysosome, occurring the cleavage of the degraded material. There are two other types of autophagy: microautophagy, with direct involvement of the material by the lysosome. In addition, there is a chaperone-mediated autophagy, encompassing the material via the LAMP-2A receptor. Together, these mechanisms improve metabolism, being an energy source through recycling amino acids and, eventually, participating in cellular quality control, which promotes an improvement in the individual lifespan and healthspan.

There are three types of autophagy currently described as macroautophagy, microautophagy, and chaperone-mediated autophagy (CMA). All differ in their mechanisms and functions [126]. Microautophagy produces random invaginations in the lysosomal membrane, involving nearby cytoplasmic components to the lysosomal lumen [127]. CMA acts directly through the lysosomal membrane via the specific receptor, LAMP-2A (lysosomal-associated membrane with protein type 2) [128]. Macroautophagy, often referred as autophagy, requires formation of a double membrane (autophagosome) involving the material to be degraded and subsequently being fused to the lysosome (Figure 6) [129].

Autophagy is mainly downregulated by the mammalian target of rapamycin (mTOR) complex. This mTOR complex is activated under nutrient-rich conditions, playing a fundamental role in the nutrient sensitivity [130]. In a nutrient-poor condition, another energetic sensor is activated, AMPK, which directly inhibits mTOR by its direct phosphorylation as well as directly activates the ULK1 (ATG protein family) (ATG, genes related to autophagy), stimulating autophagy [129]. The nutrient shortage also regulates the autophagy at the transcriptional level by modulating the expression of ATG-encoded genes, and this mechanism is mediated, at least in part, by the transcription factor FOXO1 [131].

In aging, autophagy is deregulated or inoperative, favoring the accumulation of “garbage” into the cell [132]. Overexpression of mTOR complex during aging increases abnormal protein aggregates, being related to the genesis of CVDs [133]. On the other hand, enhancing autophagy by mTOR inhibition or AMPK activation leads to an increase in healthspan, improving the cardiovascular function and prevents CVDs [134, 135]. However, its excessive autophagy activation seems to have a deleterious effect on the cardiovascular system [136]. Thereby, autophagy seems to be a compensatory effect on cellular energy levels that depend on mitochondrial dysfunction, and to understand the crosstalk between both regulators is essential to connect the energetic signalling to metabolism.

## 8. Metabolic Control of Aging: Connecting the Dots

**Figure 7:**
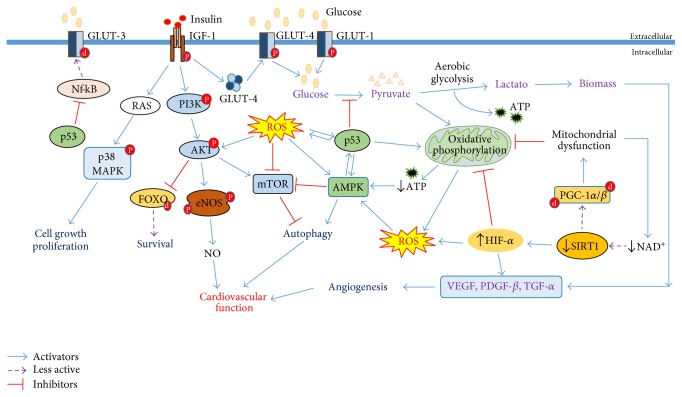
Metabolic control involved in aging and on the cardiovascular system. IGF-1, mTOR, AMPK, SIRT1, p53, and ROS are key regulators in metabolic control. Many of these pathways are complex involving crosstalk between them, with many paradoxical effects. The stimulation of the IGF-1 pathway by insulin promotes PI3K/AKT pathway activation, which induces the exclusion of FOXO from the nucleus, inhibiting its function; in addition, IGF-1 activates eNOS, increasing NO availability, improving the vascular function. IGF-1 also activates the RAS/p38MAPK pathway inducing mechanisms of cell growth and proliferation. Finally, IGF-1 stimulates vesicles containing GLUT-4 to the cell membrane, promoting the uptake of glucose, the main cellular energy substrate. There are also other two glucose transporters that help in glucose uptake such as GLUT-1 and GLUT-3; the last one can be downregulated by p53 via Nf-kB. Under normal conditions, mostly, pyruvate is directed to the mitochondria, producing ATP by OXPHOS. In age, NAD^+^ levels decrease, driving towards a loss in SIRT1 activity, resulting in mitochondrial dysfunction via PGC-1*α*/*β* and HIF-*α*. Thus, pyruvate is directed to lactate production, even in the presence of O_2_, a process known as “the Warburg effect”. This metabolic shift is essential for increasing biomass, stimulating cell growth, proliferation, and differentiation, which promote angiogenesis. In this way, a mitochondrial dysfunction results in a decreased ATP production, activating AMPK. This protein stimulates autophagy, generating energy for the cell. In addition, it stimulates p53, which inhibit the uptake and conversion of glucose, to stimulate OXPHOS activity, generating an antiproliferative effect. Finally, ROS produced by mitochondrial dysfunction stimulates several signalling pathways, such as AMPK, but also activates AKT, which stimulates mTOR, being the redox potential the major regulator of this balance.

Aging is characterized by a decrease in cellular energy supply [7]. The major regulators of this process are the mitochondria as a source of ATP and the lysosomes, an essential organelle for the autophagy, one of the mechanisms responsible for generating energy in times of nutrient scarcity [137, 138]. Several mechanisms that enhance the function of these processes play a beneficial role in lifespan and healthspan [132, 139]. The mechanism involved in this process has several regulators such as insulin/IGF-1, mTOR, AMPK, and sirtuins [140]. Other factors such as ROS and p53 pathway also appear to be part of cellular energy control (Figure 7) [141, 142].

The insulin/growth factor-1 (IGF-1) pathway controls survival, proliferation, and metabolic processes. This mechanism is one of the well-characterized pathways of lifespan, conserved from yeast to mammals [143]. Low levels of insulin and IGF-1 induced by caloric restriction (CR) or metformin are associated with improved healthspan and increased longevity [140]. Interestingly, humans with exceptional longevity present low IGF-1 [144]. This effect on lifespan is, at least in part, due to the fact that IGF-1 promotes an intracellular pathway mediated by PI3K-AKT, allowing the phosphorylation of proteins known as Forkhead box O (FOXO) [145].

The AKT-mediated phosphorylation of FOXO promotes its exclusion from the nucleus to the cytoplasm, suppressing gene transcription dependent on FOXO proteins [146]. In addition, the FOXO family is sensitive to the redox potential, being ROS levels' positive modulators for their activity [147]. The FOXO family comprises evolutionarily conserved isoforms (FOXO1, FOXO3, FOXO4, and FOXO6 in mammals, DAF-16 in *C. elegans*, and DFOXO in *D. melanogaster*), and its activity is related to various cellular processes including glucose metabolism, cell differentiation, apoptosis, DNA repair, and cellular detoxification [146, 148].

The protein kinase mTOR (mammalian target of rapamycin) is an atypical serine/threonine kinase that exerts its main cellular functions by interacting with specific adaptor proteins to form two different multiprotein complexes, mTOR complex 1 (mTORC1) and mTOR complex 2 (mTORC2) [149]. The mTOR complex is one of the major cellular regulators of nutrient sensitivity, being activated in the presence of growth factors and in abundances of cellular nutrients [130]. In aging, increased mTOR activity is linked to senescence and autophagy deficiency. Treatment with compounds, such as rapamycin delay replicative senescence, reduces senescence induced by DNA damage and reduces mitochondrial dysfunction by inhibit the mTOR complex [150, 151].

AMPK is another master regulator to cellular energy status [152]. In mammals, it is activated when the AMP/ATP and ADP/ATP ratio is elevated, which occurs when ATP production is compromised. Under this circumstance, its response has the purpose to activate alternative catabolic ATP-producing pathways, plus by inhibiting ATP-consuming processes [152, 153]. Thus, AMPK activates a series of compensatory responses including fatty acid oxidation (*β*-oxidation), inhibition of fatty acid synthesis, increased mitochondrial biogenesis, and stimulation of glucose uptake [154]. Treatment with compounds that increases AMPK levels, such as metformin, has been shown to be beneficial in longevity, insulin resistance, and increase in physical performance [155]. In addition, there is evidence that AMPK activation increases the lifespan and is related to the improvement of metabolism in mice [5]. However, how AMPK acts on aging is quite complex and still remains to be clarified.

Sirtuins (SIRTs), the homologue of silent information regulator 2 (Sir2) present in *Saccharomyces cerevisiae*, consist of a family of essential proteins for mechanisms of cell defence. These proteins require NAD^+^ for its activation [156]. In mammals, there are seven subtypes, located in different cellular compartments: nucleus (SIRT1, SIRT6, and SIRT7), cytosol (SIRT2), and mitochondria (SIRT3, SIRT4, and SIRT5) [157]. This family regulates a range of cellular events including metabolism, apoptosis, energy supply, cell survival, development, cellular differentiation, inflammation, and healthy aging [158, 159]. In aging, SIRT1 stimulates cardioprotection, inducing resistance against hypertrophic and oxidative stress, also inhibits cardiomyocyte apoptosis, and regulates cardiac metabolism [160]. SIRT1 activation induced by CR improves heart protection from ischemia/reperfusion, and this effect is abolished in SIRT1 knockout mice [161]. In addition, compounds that are able to induce SIRT1 activation, such as resveratrol, also appear to induce cardioprotection by reducing ROS production [160, 162].

The p53 protein is known to induce a range of antiproliferative processes, such as cell cycle arrest, leading to senescence and apoptosis in response to cellular stress [163]. In addition, p53 plays a critical role in monitoring and modulating cellular metabolic status, controlling, at least in part, processes such as glycolysis, oxidative phosphorylation, insulin sensitivity, mitochondrial integrity, fatty acid oxidation, and autophagy [164, 165].

p53 counteracts glycolysis by directly inhibiting the expression of GLUT1 and GLUT4 glucose transporters [166, 167] and indirectly by inhibiting GLUT3 via Nf-kB, resulting in a decrease in glucose uptake [168]. In addition, p53 controls a wide range of proteins that participate in glycolysis, acting as a glycolytic activity regulator [169, 170]. On the other hand, p53 promotes oxidative phosphorylation by inducing the expression of cytochrome c oxidase 2 (SCO2) and inhibits pyruvate dehydrogenase kinase 2 (PDK2) through parkin (PARK2), regulating mitochondrial respiration [171, 172]. Thus, p53 protein acts by connecting the cellular energy supply and senescent stage, being one of the most important regulators for the aging process [141, 173, 174]. The same mechanisms that lead to aging described above can be implicated on the cardiovascular system, being related to the balance between health and diseases, including CVDs.

## 9. Aging: Implications on the Cardiovascular System

Cardiovascular aging is defined as an age-dependent progressive degeneration, which makes the heart and vessels more vulnerable to stress, contributing to increased mortality and morbidity [175]. Notably, the vascular aging is characterized by molecular, structural, cellular, and physiological changes, being aging the main risk factor in the pathogenesis of CVDs [176, 177].

In the aged heart, several complex modifications including diastolic dysfunction, left ventricular hypertrophy, increased risk of atrial fibrillation, and valvular degeneration lead to a decreased exercise capacity, which is related to heart failure [178].

Under normal conditions, vessels have the ability to respond to various stimuli, such as vasoconstriction due to an adrenergic or circulatory (e.g., angiotensin II or endothelin II) agonist response [179]. On the other hand, vasodilator mediators such as nitric oxide (NO), endothelium-derived hyperpolarizing factor (EDHF), and some prostaglandins (e.g., PGI2) have the mission of balancing the vascular tonus [180, 181].

In fact, the production of NO is the major marker of the vascular function [182, 183]. In the vessel, its synthesis is made mainly by endothelial nitric oxide synthase (eNOS), being aging associated to a decrease in the NO production [184–186]. In senescent-accelerated mice, endothelial dysfunction associated with aortic age is linked to eNOS dysfunction [187]. Increased release of ROS and subsequent inactivation of NO are important mechanisms involved on the impairment of endothelium-dependent vessel relaxation, leading to stiffness and vascular inflammation [188, 189].

The vascular aging leads to thickening of the intima and media layer (vascular remodelling), as well as gradual loss of arterial elasticity, resulting in vascular rigidity [190, 191]. Increased collagen and decreased elastin content, promoted at least in part by age, in addition to increased glycosylated proteins, matrix metalloproteinase activity, and systemic stimuli such as angiotensin II signalling, are linked to vascular rigidity [192, 193].

**Figure 8:**
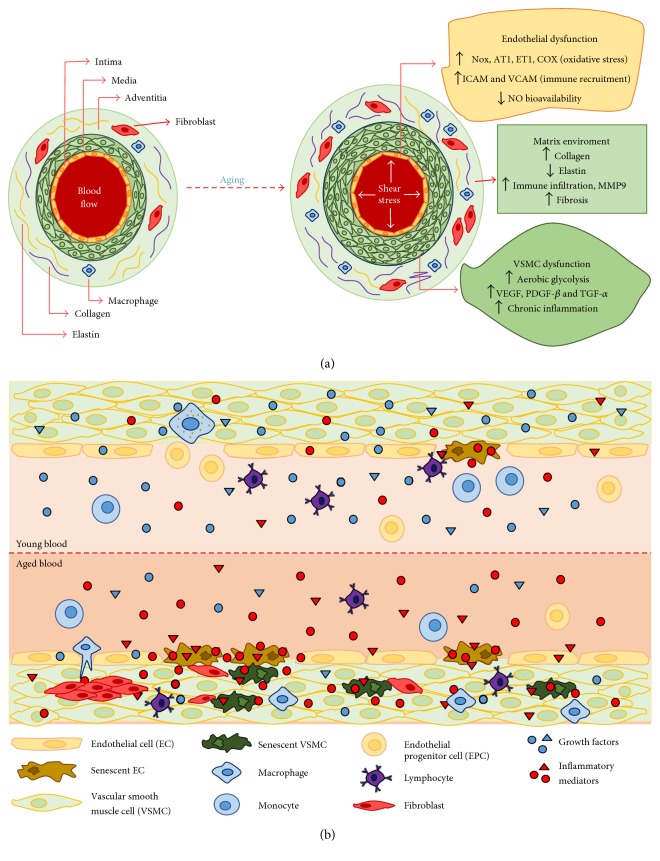
Young and aged vascular comparison in two different perspectives. In vascular aging, the remodelling occurs due to the accumulation of senescent and dysfunctional cells in response to the environmental changes caused by age. (a) In the aged vessel, there is a loss of the vessel elasticity, due to the raises of contracting factors, plus an increase in the number of muscle cells. These factors combined drive towards the matrix change, with inflammatory infiltrates and fibrosis, leading to vascular hypertrophy. (b) In young blood, there is a predominance of growth factors, in addition to healthy cells of immunity and progenitor cells driving towards vascular “cleaning” and regeneration, respectively. In the aged blood, it is checked that there is a predominance in proinflammatory factors, released largely by senescent cells. The senescent cells accumulate with age in response to a failure of the immune system, a term known as immunosenescence. In addition, there is an increase in fibroblast proliferation, leading to a stressful environment related to the vascular remodelling.

Aged endothelial cells (ECs) and VSMCs also show increased secretion of proinflammatory cytokines, derived in large part from senescent cells, which results in persistent vascular inflammation [30, 101]. In addition, VSMCs change their metabolic route to promote aerobic glycolysis (in response to mitochondrial dysfunction), being essential to produce a high rate of substrate for cellular growth and proliferation, and to express factors such as vascular endothelial growth factor (VEGF), platelet-derived growth factor (PDGF-*β*), and transforming growth factor alpha (TGF-*α*) that contribute to the vascular remodelling (Figure 8(a)) [194, 195].

The vasculature also plays an important role in connecting all the tissues through the blood flow. In fact, the vascular inflammation extends to other organism components leading to a systemic effect [196]. In the young blood, there is a predominance of growth factors in detriment of inflammatory mediators, plus healthy immunity cells and endothelial progenitor cells, which are essential for vascular “cleaning” and regeneration [197]. On the other hand, aged blood has predominance of proinflammatory factors, largely released by senescent cells [198]. In addition, there is a failure of the immune system, resulting in the accumulation of senescent cells in the vascular tissue, leading to a stressful environment, which is associated with the development and progression of CVDs [196, 199].

An elegant study performed by Loffredo and colleagues [200] demonstrated that changing the systemic influence from the blood by connecting young to aged blood by parabiosis (surgical technique that unites the vasculature of two living animals) showed that after 4 weeks, aged rats that were exposed to young circulation had reversed age-related cardiac hypertrophy, resulting in cardiovascular protection.

Thus, the vascular remodelling, by aging or pathological conditions, is accompanied by oxidative stress and inflammation, leading to an increase of senescent cells in these tissues (Figure 8(b)) [59, 201]. The endothelial cells have fundamental importance in the development of vascular remodelling, being an endothelial dysfunction target of therapies against CVDs, such as hypertension, atherosclerosis, and heart failure [202–204]. Treating aging seems to show several benefits on the cardiovascular system, by creating a healthy systemic environment, which slows the progression of endothelial dysfunction and the vascular remodelling associated with aging, leading to cardiovascular protection.

## 10. Conclusion and Future Directions

In this review, we discuss cellular mechanisms related to aging. It is possible to notice that aging is a multifactorial process that encompasses intrinsic factors to several species and the accumulation of senescent cells is common in the main part of them. Understanding the aging process, we may find the genesis of age-related diseases, since many of them are characterized by disorders that are consequences of cellular dysfunction caused by senescence. This accumulation of senescent cells can have a replicative genesis, bringing into action therapeutic targets such as telomerase, as well as induced by stress, such as the cellular energetic loss, which encompasses the mitochondria dysfunction and deregulated autophagy. These mechanisms are connected by a series of proteins, transcription factors, and environmental factors into the cell, such as redox potential. However, a determinant factor controlling the whole process remains unclear. One of the candidates would be to understand how the redox potential determines gene expression and promotes responses in metabolism. The fact that ROS promotes an increase in redox potential and this hallmark is involved in aging as well as in age-related diseases makes us believe that the increase in cellular ROS is intentional by the cells, in order to promote cellular survival mechanisms, requiring more and more ROS to have the same effect over time, a process that drives towards the deleterious effects of ROS. Understanding how the concentration and localization of ROS and its interaction with longevity genes may be a key point to understand the complex metabolic mechanism that controls aging. In this way, it will be possible, in the future, to take a pill that promotes an increase in longevity and, in addition, play a role in minimizing the onset of aging-related diseases.
